# Modeling the normal:abnormal spectrum of early childhood internalizing behaviors: A clinical‐developmental approach for the Multidimensional Assessment Profiles Internalizing Dimensions

**DOI:** 10.1002/mpr.1987

**Published:** 2023-10-10

**Authors:** Lauren S. Wakschlag, Phillip Sherlock, Courtney K. Blackwell, James L. Burns, Sheila Krogh‐Jespersen, Richard C. Gershon, David Cella, Kristin A. Buss, Joan L. Luby

**Affiliations:** ^1^ Department of Medical Social Sciences Feinberg School of Medicine Northwestern University Chicago Illinois USA; ^2^ The Institute for Innovations in Developmental Sciences Northwestern University Chicago Illinois USA; ^3^ Department of Psychology Pennsylvania State University University Park Pennsylvania USA; ^4^ Human Development & Family Studies Pennsylvania State University University Park Pennsylvania USA; ^5^ Washington University School of Medicine St. Louis Missouri USA

**Keywords:** developmental psychopathology, early childhood, internalizing problems, RDoC

## Abstract

**Background:**

We expanded the Multidimensional Assessment Profiles (MAPS) Scales developmental specification model to characterize the normal:abnormal spectrum of internalizing (anxious and depressive) behaviors in early childhood via the MAPS‐Internalizing (MAPS‐INT) scale.

**Methods:**

The MAPS‐INT item pool was generated based on clinical expertise and prior research. Analyses were conducted on a sub‐sample of families (*n* = 183) from the diverse When to Worry early childhood sample.

**Results:**

Normal:abnormal descriptive patterns for both anxious and depressive behaviors were consistent with prior work: (1) extremes of normative variation are abnormal when very frequent; and (2) pathognomonic indicators that most children do not engage in and are abnormal, even if infrequent. Factor analysis revealed a two‐factor MAPS‐INT Anxious Behaviors structure (Fearful‐Worried and Separation Distress) and a unidimensional MAPS‐INT Depressive Behaviors factor with good fit and good‐to‐excellent test‐retest reliability and validity.

**Conclusions:**

We characterized the normal:abnormal spectrum of internalizing behaviors in early childhood via the MAPS‐INT. Future research in larger representative samples can replicate and extend findings, including clinical thresholds and predictive utility. The MAPS‐INT helps lay the groundwork for dimensional characterization of the internalizing spectrum to advance neurodevelopmental approaches to emergent psychopathology and its earlier identification.

**Abbreviations**
CBCL: Child Behavior Checklist
CFA: Confirmatory Factor Analysis
CFI: comparative fit index
CI: confidence interval
DSM‐5: Diagnostic and Statistical Manual of Mental Disorders, 5th edition
ECHO: Environmental Influences on Child Health Outcomes Consoritum
ICC: intra‐class correlation coefficient
IRT: item response theory
ITSEA: Infant Toddler Social Emotional Assessment scale
MAPS Study: Multidimensional Assessment of Preschoolers Study
MAPS‐INT: Multidimensional Assessment Profiles Scales‐Internalizing Dimensions
RDoC: NIMH Research Domain Criteria
RMSEA: root mean square error of approximation
SD: standard deviation
SDQ: Strengths and Difficulties Questionnaire
SRMR: standardized root mean squared residual
TLI: Tucker‐Lewis index
ULSMV: unweighted least squares mean variance
W2W: When to Worry

## 1 INTRODUCTION

Clinical phenomena are increasingly conceptualized as traits that vary along normal: abnormal dimensions, can be identified at the neurodevelopmental vulnerability stage, and manifest as psychopathology in their extreme, persistent, frequent and impairing forms (Casey et al., 2014; Wakschlag et al., 2018; Wiggins, Ureña Rosario, Zhang, et al., 2023). Within traditional nosologic systems (e.g., DSM‐5), symptoms often overlap with normative extremes of early childhood, and many young children who exhibit precursor risk patterns do not have maladaptive outcomes (Buss & McDoniel, 2016; Luby et al., 2002; Wakschlag et al., 2010). These precursor patterns are atypical (based on psychometric distributions, dysregulation and/or occurrence in developmentally unexpectable contexts, see (Buss, 2011; Wakschlag et al., 2010)), but can be fleeting and/or not associated with impairment over time (Wiggins, Ureña Rosario, MacNeill, et al., 2023). As a result, measuring clinical constructs as unfolding dimensional risk spectra is of particular importance for defining the boundaries between normative variation and/or transient elevations, and behavioral risk markers for sustained and impairing problems within the developmental context of early childhood (Buss, 2011; Cole et al., 2008; Wakschlag et al., 2010).

To operationalize this dimensional approach in our prior work, we introduced a developmental specification approach to establish abnormal patterns of disruptive behavior as deviation from expectable variation within early childhood (Wakschlag et al., 2010). The resultant Multidimensional Assessment Profile of Disruptive Behavior (MAP‐DB) scales characterize core dimensions of disruptive behavior, including the dimension most salient to internalizing syndromes, that is, irritability (for details see, Nichols et al., 2014; Nili et al., 2021; Wakschlag et al., 2014; Krogh‐Jespersen et al., 2021; Wakschlag et al., 2018). (*Note*: While originally termed the MAP‐DB, this suite of scales has now been renamed the MAPS Scales. This reflects the expansion of this dimensional approach to attentional regulation (Nili et al., 2023), and now internalizing, syndromes). In this prior work, features critical to normal:abnormal differentiation were behavioral dysregulation and developmental unexpectability in context (i.e., likelihood that particular stimuli or contexts typically elicit this behavior in an age period). This uncovered two key patterns: (1) extremes of normative variation such that the behaviors occur in the majority of children and are only atypical when very frequent. For this distinction, we define “regularly” as behaviors that occur monthly in >50% of children (e.g., having a tantrum with a parent) (Wakschlag et al., 2018); and (2) pathognomonic behaviors in which the behavior does not typically occur within the realm of normative variation and its occurrence is a red flag. For all behaviors, item response theory (IRT) psychometric threshold of abnormality was set at the frequency at which behaviors occurred in <5% of children (Wakschlag et al., 2018). The utility of this dimensional psychometric approach has been extensively demonstrated for irritability (Damme et al., 2022; Grabell et al., 2017; Krogh‐Jespersen et al., 2021; Wiggins, Ureña Rosario, MacNeill, et al., 2023; Wiggins, Ureña Rosario, Zhang, et al., 2023; Hirsch et al., 2023; Alam et al., 2023; Kirk et al., 2023; Wiggins, Ureña Rosario, MacNeill et al., 2023).

Our approach also draws on foundational research on temperament (Biederman et al., 2001; Chronis‐Tuscano et al., 2009; Rothbart et al., 2011). Temperament theory is undergirded by the principle that expression of negative affect may be adaptive, and that taking into account patterns and contextual features of its regulation is critical for differentiating normative variation from clinical risk (Buss & McDoniel, 2016; Cole et al., 2008). Fundamental to this approach is that individual variability in negative affect and regulation are both normative (for most children), but also signal risk for psychopathology at the extremes (Ostlund et al., 2021). For example, accounting for dysregulated fear observed in contexts that do not typically elicit fear improves prediction of which fearful toddlers will develop anxiety disorder symptoms (Buss, 2011; Buss et al., 2013). Similarly, diminished positive emotion in response to pleasant events is a context‐inappropriate emotional expression of salience to early depression (Cole et al., 2008). Emphasis on occurrence in developmentally expectable versus unexpectable contexts is a unique feature of the MAPS dimensional framework.

Decades of research supports the importance of temperament on adaptive and maladaptive outcomes. Behavioral inhibition in toddlers‐ a temperament type characterized by extreme fear, wariness, and avoidance of novelty (Coll et al., 1984)‐ is the best early predictor of social anxiety across childhood and into adolescence (Clauss et al., 2015). Moreover, behavioral inhibition and later avoidance can be reliably predicted from a pattern of negativity reactivity as early as 4 months (e.g., Hane et al., 2008; Kagan & Snidman, 1991a, 1991b); and negative affect has also been linked to anxiety symptoms even early in development (Buss et al., 2021). Likewise, temperamental vulnerability for depression is also a robust finding in the literature with low positive emotionality and high negative emotionality serving as early risk factors for the development of depressive symptoms (Dougherty et al., 2010; Nielsen et al., 2019; Stifter & Dollar, 2016). In essence, the developmental literature has established that temperamental extremes, when persisting over time, represent a risk factor for forms of internalizing psychopathology. However, to date, there has not been a standardized, efficient parent‐report measure specifically designed to capture the normal: abnormal spectrum of internalizing behaviors (as temperament measures are designed to capture normative variation) in a manner that meaningfully captures precursor pathways. Further, historically, there has been a reluctance to label precursor behaviors as clinical red flags despite evidence of that they reliably indicate increased likelihood of impairing psychopathology. This has reflected concerns about stigma and over‐pathologizing and preference for “watching and waiting” with the hope that these behaviors are developmentally transient (Luby, 2012). There is increasing evidence of the safety and efficacy of early interventions for both depression and anxiety (Fisak et al., 2023; Luby et al., 2020; Morgan et al., 2017). Based on this, earlier identification and prevention of these precursor patterns through the lens of developmental psychopathology when appropriate is warranted (Clauss et al., 2015; Jerome & Snidman, 1991b).

Recent methodologic work by Bufferd, Dougherty and Olino has built a critical bridge between developmental and clinical understanding of these early internalizing patterns within a dimensional developmental specification framework (Bufferd et al., 2017, 2019, 2023). Using daily diary reports of objective frequency of behavioral occurrence to differentiate typical and atypical manifestations of internalizing behaviors in early childhood, they are the first to characterize the dimensional spectrum of these behaviors. Consistent with our prior work in the disruptive behavior domain (Wakschlag et al., 2014), they have demonstrated that differentiation of typical:atypical internalizing behaviors can be modeled along a severity continuum (Bufferd et al., 2023), and can be distinguished in terms of frequency of occurrence (which varies based on the nature of behavior) and context (Bufferd et al., 2017, 2019).

Building on this convergent evidence base, the Multidimensional Assessment Profiles ‐ Internalizing (MAPS‐INT) Scales measure was designed to characterize the dimensional spectra of anxious and depressive behavior across the early childhood period (ages 1–5 years) within a survey form. Validation of pragmatic, developmentally based survey methods is key for utilization in large epidemiologic studies and in real world clinical settings (Morris et al., 2020; Wakschlag et al., 2022). We descriptively examine whether normal:abnormal differentiation in these internalizing behaviors mirrors what has been previously reported, and model anxious and disruptive behavior dimensions with the MAPS‐INT.

## 2 METHODS

### 2.1 Overview

Data are drawn from a sub‐study of the When to Worry (W2W) Study, a study of infant irritability as an early marker of psychopathology risk (Krogh‐Jespersen et al., 2021; Zhang et al., 2023). This survey sub‐study psychometrically validates the MAPS‐INT. Data were collected in Summer 2019.

### 2.2 Participants

Participants from the W2W sample were invited by email to participate in a survey sub‐study. The W2W sample (*N* = 356) is diverse and was ascertained in the greater Chicago region with oversampling for irritability (for details, see Krogh‐Jespersen et al., 2021; Zhang et al., 2023). One hundred and eighty‐three participants completed the survey. The Northwestern University Institutional Review Board approved all study procedures.

To obtain the largest possible sample within the ongoing W2W study for this survey sub‐study, the MAPS‐INT sub‐sample includes a broad range of ages spanning early childhood (*M* = 30.3 months, *SD* = 6.0, range = 13.7–44.5 months). The survey sub‐sample was comparable to the remaining W2W sample (*n* = 173) on age, gender, and baseline irritability. However, the sub‐sample included more non‐Hispanic white and non‐poor children (see Supplementary Table S1).

### 2.3 Measures

#### 2.3.1 Multidimensional Assessment Profile‐Internalizing (MAPS‐INT) Dimensions

Drawing on our extensive foundational work, the MAPS‐INT scales for anxious and depressive behaviors were developed by the authors who are psychometric scientists, and developmental psychologists/psychopathologists. This includes the developmental specification theory and psychometric approach underlying the generation and validation of the MAPs irritability “Temper Loss” scale (Wakschlag et al., 2014; Wakschlag et al., 2010; see also Alam et al., 2023; Hirsch et al., 2023; Kirk et al., 2023; Wiggins, Ureña Rosario, MacNeill, et al., 2023), and a large body of work on the developmental phenotype of anxious and depressive temperamental and clinical patterns (Buss, 2011; Buss & McDoniel, 2016; Cole et al., 2008; Luby et al., 2003). We generated an item pool to capture varied developmental expressions along a continuum of dysregulation and contextual expression that varied in terms of developmental expectability. There were 34 items in the anxious behavior pool and 28 in the depressive behavior pool (Supplementary Tables S2–S3). To enhance normal:abnormal differentiation and reduce bias emanating from variations in parental tolerance for misbehavior, items on the MAPS scales are rated on a 6‐point objective frequency scale: 0 = never; 1 = rarely (<1x/week); 2 = some days (1–3) of the week; 3 = most days (4–6) of the week; 4 = daily; and 5 = many times each day. Consistent with the validated MAPS measure (Wakschlag et al., 2014), parents used these anchors to reply to the query “Over *the past month, how often did your child*…?” This 30 days reporting interval was chosen based on best practices for choice of recall period in survey measures (Norquist et al., 2012). Specifically, we balanced considerations of reliability of memory for recall interval, with the need to ensure that behaviors captured are not fleeting in this period of rapid developmental change.

#### 2.3.2 Validation surveys

To validate the MAPS‐INT scales, we used three well‐validated scales: (1) the Anxious/Depressed, Somatic Complaints, Withdrawn, and Internalizing scales of the Child Behavior Checklist (CBCL) (Achenbach & Rescorla, 2000); (2) the Emotional Problems and Prosocial Behavior scales of the Strengths and Difficulties Questionnaire (SDQ) (Goodman & Goodman, 2009), and the Inhibition to Novelty, Separation Distress, Anxiety and Depression/Withdrawal scale of the Infant‐Toddler Social‐Emotional Assessment (ITSEA) (Briggs‐Gowan & Carter, 1998).

### 2.4 Analytic approach

To test whether internalizing problems in young children manifest two behavioral patterns as extremes of normative variation versus pathognomonic, we first examined whether objective frequency distribution distinguished the former from the latter. To examine the factor structure of the MAPS‐INT, we then conducted confirmatory factor analysis using Mplus Version 8.4 (Muthén & Muthén, 2019). We used the unweighted least squares estimator with robust standard errors and mean‐ and variance‐adjusted Chi‐square test statistic (ULSMV).

Model fit was guided by best practices (i.e., the root mean square error of approximation (RMSEA) and its 90% confidence interval, the comparative fit index (CFI), the Tucker–Lewis index (TLI), and the standardized root mean square residual (SMRI) (SMRI, Kline, 2016)). Acceptable model fit was defined as follows: RMSEA (≤0.06, 90% CI ≤ 0.06), CFI (≥0.95), TLI (≥0.95), and SRMR (≤0.08). Model specification was also evaluated based on factor loadings and modification indices. Two‐week factor test‐retest reliability was examined with rank‐based intra‐class correlation coefficients (ICCs) (*n* = 116). ICC values were evaluated based on the following cutoffs: below 0.40 (poor); 0.40–0.75 (good); and above 0.75 (excellent) (Fleiss, 1986).

We examined convergent/divergent validity by calculating correlations between MAPS‐INT dimensions and the CBCL, SDQ and ITSEA scales (Achenbach & Rescorla, 2000; Goodman & Goodman, 2009; Briggs‐Gowan & Carter, 1998).

## 3 RESULTS

### 3.1 Developmental patterning of young children's anxious behaviors

Consistent with prior work within the developmental specification framework, behaviors tended to fall in two normative and pathognomonic broad patterns (see Table S2 for item level distributions). First, there were a set of anxious behaviors displayed by most children; empirically defined as those that 50% or more young children are reported to do regularly. Seven of the anxious behaviors fell into this group (including separating from parent in various contexts, acting worried when trying new things, and being scared or fearful). Roughly two‐thirds of parents reported that their young children engaged in this set of behaviors over the course of the past month, with 21%–35% of parents reporting these occurred weekly. These common behaviors became psychometrically severe (i.e., fell in the atypical range) if they occurred very frequently. Second, the remaining 28 items were consistent with previously established pattern of pathognomonic indicators (i.e., behaviors that have not occurred in most children over the past month, and mark atypicality by their relatively rare occurrence). These include a number of dysregulated behaviors (e.g., inconsolability, keep worrying despite reassurance, freezing) and becoming fearful and anxious in developmentally unexpectable contexts (e.g., familiar settings). For example, only 10% of parents reported that their young children had become *uncontrollably distressed when anxious* and only 14% reported that their young children had *suddenly become anxious out of nowhere* over the past month. In general, less than 10% of children exhibited any normative misbehaviors or pathognomonic indicators many days per week (i.e., 4–6 days or more). The exceptions were 3 separation related items—*act worried when separating*, *clinging when separating in an unfamiliar setting*, and *clinging when separating even after reassurance*. These were endorsed for 11%–14% of children. Daily occurrence of this behavior was very rare (≤5%).

### 3.2 Anxious behavior factor Model (CFA)

We used all administered items to conduct a two‐factor confirmatory factor analysis for anxious behaviors. We distinguished fear and distress factors, based on prior work indicating distinct clinical and mechanistic pathways (Dougherty et al., 2013; Edwards et al., 2010; Kendler et al., 2003; Rapee & Coplan, 2010; Salum et al., 2013). Analyses yielded *Fearful‐Worried* and *Separation Anxiety* factors, which also map to DSM distinctions between general anxiety disorder (GAD) and social/relational patterns such as separation anxiety disorder (SAD) (Clauss et al., 2015; Kagan & Snidman, 1991b). The modification index suggested that two sleep‐related items—*Seem fearful or worried at bedtime* and *wake up at night scared*—should have correlated residuals, which we added to the final model.

Overall, the final model had good fit. Each of the other a priori chosen fit indices suggested that a two‐factor Anxious Behaviors model fit the data well, RMSEA = 0.05 (90% CI = 0.05–0.06), CFI = 0.96, TLI = 0.96, and SRMR = 0.08. Delta‐scaled, fully standardized (i.e., STDYX) parameter estimates from the final model are presented in Table 1 (Fearful‐Worried and Separation Distress). All standardized factor loadings were significant (*p* < 0.001), with indicators moderately to strongly related to their respective latent factors. Fearful‐Worried and Separation Distress were moderately correlated (0.68), suggesting good discriminant validity. Good test‐retest reliability was demonstrated; ICC values for the Fearful‐Worried and Separation Distress factors were 0.83 (95% C.I. = [0.76, 0.88]) and 0.71 (95% C.I. = [0.61, 0.79]), respectively.

**TABLE 1:** Standardized loadings (standard errors) and threshold probabilities for the Anxious Behaviors Factors.

Item	*λi*	Never	<Weekly	1–3 days/week	1–3 days/week	Daily
Fearful‐worried						
Seem scared or fearful	0.75 (0.04)	0.37	0.78	0.96	0.99	0.99
Act worried when meeting new people/trying new things	0.76 (0.04)	0.46	0.79	0.94	0.99	
Seem worried	0.78 (0.04)	0.59	0.85	0.98	0.99	
Wake up at night scared	0.48 (0.06)	0.61	0.86	0.97		
Seem nervous	0.82 (0.04)	0.62	0.89	0.99	0.99	
Seem fearful or worried when out in public	0.86 (0.03)	0.62	0.90	0.98	0.99	
Seem fearful or worried at daycare, school, or other familiar settings away from home	0.71 (0.05)	0.63	0.90	0.99	0.99	
Get startled easily	0.66 (0.05)	0.64	0.88	0.97	0.99	
Remain inconsolable even after receiving reassurance	0.62 (0.05)	0.64	0.92	0.99	0.99	
Keep worrying even after receiving reassurance	0.86 (0.04)	0.66	0.90	0.99		
Get scared really easily	0.78 (0.04)	0.67	0.89	0.98	0.99	
Seem afraid to engage in things that are fun	0.79 (0.04)	0.68	0.93	0.99		
Seem anxious	0.77 (0.04)	0.69	0.90	0.98	0.99	
Seem fearful or worried at home	0.80 (0.04)	0.72	0.94	0.99		
Seem fearful or worried at bedtime	0.70 (0.04)	0.74	0.90	0.97	0.99	
Act worried when out with you or other parent in public	0.83 (0.04)	0.74	0.95	0.99		
Seem fearful or worried during fun activities	0.87 (0.03)	0.75	0.96			
Seem tense	0.73 (0.05)	0.75	0.95			
Act worried when in a group of children	0.85 (0.04)	0.78	0.91	0.98		
Worry about what could happen to him/her	0.78 (0.05)	0.78	0.96	0.99		
Act scared before daycare/preschool	0.62 (0.07)	0.79	0.92	0.97	0.99	
Act worried during daily routines, such as bedtime, mealtime, or getting dressed	0.91 (0.03)	0.80	0.95			
Suddenly become anxious “out of nowhere” or for no reason	0.89 (0.04)	0.86	0.96	0.99		
Freeze because he or she was so scared	0.80 (0.05)	0.87	0.98	0.99		
Become uncontrollably distressed when anxious	0.75 (0.06)	0.90	0.98			
Separation distress factor						
Cling even after receiving reassurance when separating from you or other parent	0.80 (0.04)	0.34	0.66	0.87	0.95	0.98
Cling when separating from you or other parent in an unfamiliar setting (e.g., new school, new friend's house)	0.78 (0.05)	0.34	0.70	0.86	0.95	0.98
Cling when separating from you or other parent in a familiar setting (e.g., familiar daycare/babysitter)	0.72 (0.05)	0.37	0.76	0.91	0.97	0.99
Cling when separating from you or other parent at home	0.76 (0.05)	0.40	0.71	0.91	0.96	0.98
Act worried when separating from you or other parent	0.94 (0.03)	0.42	0.75	0.89	0.97	0.99
Become inconsolable when separating from you or other parent in a familiar setting (e.g. familiar daycare/babysitter)	0.82 (0.04)	0.55	0.82	0.92	0.96	
Become inconsolable when separating from you or other parent in an unfamiliar setting (e.g., new school, new friend's house)?	0.85 (0.04)	0.58	0.77	0.91	0.96	0.99
Become inconsolable when separating from you or other parent at home	0.79 (0.05)	0.58	0.82	0.94	0.97	0.99
Act afraid to stay at birthday party or playdate by themselves	0.83 (0.06)	0.70	0.86	0.95	0.98	

### 3.3 Developmental patterning of young children's depressive behaviors

The distributions of Depressive Behaviors items are shown in Supplementary Table S3. Consistent with patterns described above, behaviors were distributed as extremes of normative variation, and pathognomonic patterns (Table S3). First, six Depressive Behaviors displayed extremes of normative variation pattern occurring in more than 50% of young children (e.g., *seem sad*, *get tearful during daily routines*). Roughly two‐thirds of parents reported that their young children engaged in this set of behaviors over the course of the past month, with 18%–29% of parents reporting that these occur on a weekly basis (*not seem to enjoy activities/play* was an exception, occurring in more than 50% of children but only 8% of parents endorsed this as occurring on a weekly basis). These common behaviors become abnormal if they occur very frequently. Second, the remaining 22 items were consistent with our established pattern of pathognomonic indicators (i.e., behaviors that have not occurred in most children over the past month, and mark atypicality). These include a number of dysregulated, intense expressions of depressive behavior (e.g., *being too sad to eat, acting mopey throughout the day, lacking energy, and acting sad*) or withdrawn in developmentally unexpectable contexts (e.g., *not as excited as you would expect, sad when playing with other kids*). For example, only 8%–9% of parents reported that their young children *said negative things about themselves* and were *too sad to eat* over the past month. In general, less than 10% of children exhibited any normative misbehaviors or pathognomonic indicators multiple days per week (i.e., 4‐6x/week or >), and daily occurrence was very rare (<5%).

### 3.4 Depressive Behavior Factor Model (CFA)

The initial single‐factor CFA for depression was estimated using all the administered items. (Note: Because irritability is captured in a separate transdiagnostic dimension in MAPS‐Temper Loss [TL], the Depressive Behaviors scale only captures non‐irritable depressive phenomenology—see discussion.) Modification indices did not suggest the addition of any paths. However, the low‐standardized factor loadings associated with four pairs of related items prompted the removal of one item per pair. Consequently, the following items with smaller R‐squared values in their respective pairs, were removed from the final model (i.e., *keep crying even when you or other parent tried to comfort him/her*; *act sad or gloomy*; *lack enjoyment when interacting with unfamiliar adults*; and *lack enthusiasm*). Each of the *a priori* chosen fit indices suggested that the single‐factor Depressive Behavior model fit the data well, RMSEA = 0.05 (90% CI = 0.04–0.06), CFI = 0.97, TLI = 0.96, and SRMR = 0.08. Delta‐scaled, fully standardized (i.e., STDYX) parameter estimates from the final model are presented in Table 2. All standardized factor loadings were statistically significant (*p* < 0.001). Standardized factor loading estimates revealed that the indicators moderately to strongly related to the hypothesized latent factor. Based on the results from the ICC calculation, the Depressive Behavior scale displayed excellent test‐retest reliability with an ICC value equal to 0.86 (95% C.I. = [0.81, 0.90]).

**TABLE 2:** Standardized loadings (standard errors) and threshold probabilities for the Depressive Behaviors Factor.

Item	*λi*	Never	<Weekly	1–3 days/week	1–3 days/week	Daily
Seem sad	0.49 (0.06)	0.29	0.75	0.95	0.98	0.99
Get tearful or weepy during daily routines, such as bedtime, mealtime, or getting dressed	0.43 (0.07)	0.40	0.71	0.94	0.98	
Act withdrawn when interacting with unfamiliar adults (e.g., store clerk, doctor)	0.62 (0.05)	0.41	0.74	0.93	0.96	0.97
Not seem to enjoy activities and play	0.67 (0.04)	0.48	0.92	0.98	0.98	
Seem uninterested in eating food he/she usually likes	0.40 (0.07)	0.53	0.84	0.98	0.99	
Not seem to enjoy interacting with other children	0.71 (0.05)	0.56	0.91	0.99		
Have a hard time having fun	0.80 (0.04)	0.56	0.97			
Not seem interested in doing things he/she usually likes	0.74 (0.05)	0.59	0.94			
Not get as excited as you or other parent would expect	0.77 (0.05)	0.61	0.93			
Act withdrawn when in a group of children	0.81 (0.04)	0.62	0.89	0.98		
Have a hard time enjoying him/herself	0.80 (0.04)	0.63	0.96			
Act withdrawn when out with you or other parent in public	0.77 (0.04)	0.64	0.94	0.98	0.99	
Not seem to enjoy interacting with other familiar adults (e.g., teacher, babysitter, family member)	0.84 (0.03)	0.64	0.95	0.99		
Not seem to enjoy interacting with you or other parent at home	0.79 (0.04)	0.67	0.98			
Seem withdrawn	0.73 (0.06)	0.68	0.94			
Act mopey throughout the day	0.66 (0.06)	0.68	0.94	0.99		
Act withdrawn when interacting with other familiar adults (e.g., teacher, babysitter, family member)	0.87 (0.03)	0.70	0.94	0.98		
Act withdrawn during daily routines, such as bedtime, mealtime, or getting dressed	0.77 (0.04)	0.75	0.95			
Suddenly act sad or gloomy “out of the blue” or for no reason	0.77 (0.05)	0.75	0.93	0.99	0.99	
Act withdrawn when interacting with you or other parent at home	0.79 (0.05)	0.77	0.98			
Lack energy and seem not to care	0.73 (0.05)	0.80	0.96	0.99		
Seem sad when playing with other kids	0.76 (0.05)	0.82	0.98			
Seem too sad to eat	0.66 (0.07)	0.91	0.99			
Say negative things about him/herself	0.47 (0.12)	0.92	0.99			

### 3.5 Convergent/divergent validity of the Anxious and Depressive Behavior Factors

Table 3 contains the correlations between the MAPS‐INT Anxious and Depressive Behaviors scales and subscales from the CBCL, SDQ, ITSEA. The MAPS‐INT Fearful‐Worried scale was most highly correlated with the Anxious‐Depressed and Internalizing scales from the CBCL, the Emotional Problems scale from the SDQ, and the Inhibition to Novelty and Anxiety scales from the ITSEA. As expected, the MAPS‐INT Separation Distress core was correlated with ITSEA Separation Distress and Inhibition to Novelty scales and the MAPS‐INT Depressive Behaviors Scale was associated with the CBCL Anxious/Depression, Somatic and Withdrawn Scales and the ITSEA Depression/Withdrawal Scale. All MAPS‐INT scales were associated with CBCL Internalizing and SDQ Emotional Problems Scales. The negative association of the SDQ Prosocial Behavior Scale with MAPS‐INT Depressive Behavior and a trend in this direction for Separation Distress provided modest evidence of divergent validity.

**TABLE 3:** Convergent/divergent validity of MAPS‐INT Anxious and Depressive Behaviors Factors.

Pearson correlation coef. (prob > |*r*| under H0: Rho = 0)	MAPS‐INT			CBCL	
	Fearful‐worried	Separation distress	Depressive behaviors	Anxious/depressed	Somatic complaints
MAPS‐INT					
Fearful‐worried	1.00				
Separation distress	0.59	1.00			
	<0.0001				
Depressive behaviors	0.67	0.42	1.00		
	<0.0001	<0.0001			
CBCL					
Anxious/depressed *T*‐score	0.44	0.44	0.29	1.00	
	<0.0001	<0.0001	<0.0001		
Somatic complaints *T*‐score	0.26	0.19	0.27	0.51	1.00
	0.0005	0.0097	0.0003	<0.0001	
Withdrawn *T*‐score	0.22	0.20	0.33	0.58	0.51
	0.0022	0.0074	<0.0001	<0.0001	<0.0001
Internalizing *T*‐score	0.51	0.44	0.48	0.66	0.67
	<0.0001	<0.0001	<0.0001	<0.0001	<0.0001
SDQ					
Emotional problems	0.49	0.40	0.29	0.52	0.33
	<0.0001	<0.0001	<0.0001	<0.0001	<0.0001
Prosocial behavior (*n* = 183)	−0.11	−0.14	−0.24	−0.19	−0.13
	0.1273	0.0624	0.0010	0.0093	0.0708
ITSEA					
Inhibition to novelty (*n* = 183)	0.43	0.42	0.34	0.37	0.18
	<0.0001	<0.0001	<0.0001	<0.0001	0.0132
Separation distress (*n* = 170)	0.37	0.69	0.28	0.36	0.21
	<0.0001	<0.0001	0.0002	<0.0001	0.0050
Anxiety (*n* = 183)	0.41	0.28	0.24	0.49	0.55
	<0.0001	<0.0001	0.0009	<0.0001	<0.0001
Depression/withdrawal	0.26	0.11	0.26	0.59	0.48
	0.0004	0.1518	0.0004	<0.0001	<0.0001

	CBCL		SDQ		ITSEA
	Withdrawn	Internalizing	Emotional problems	Prosocial behavior	Inhibition to novelty
CBCL					
Withdrawn *T*‐score	1.00				
Internalizing *T*‐score	0.61	1.00			
	<0.0001				
SDQ					
Emotional problems	0.26	0.46	1.00		
	0.0004	<0.0001			
Prosocial behavior (*n* = 183)	−0.28	−0.20	−0.03	1.00	
	0.0001	0.0061	0.7210		
ITSEA					
Inhibition to novelty (*n* = 183)	0.21	0.43	0.42	−0.14	1.00
	0.0041	<0.0001	<0.0001	0.0676	
Separation distress (*n* = 170)	0.23	0.43	0.29	−0.16	0.45
	0.0031	<0.0001	<0.0001	0.0418	<0.0001
Anxiety (*n* = 183)	0.37	0.62	0.40	0.08	0.29
	<0.0001	<0.0001	<0.0001	0.2742	<0.0001
Depression/withdrawal	0.65	0.45	0.25	−0.26	0.12
	<0.0001	<0.0001	0.0006	0.0005	0.1051

## 4 DISCUSSION

A central tenet of developmental psychopathology is conceptualization of clinically salient patterns as deviations from normative patterns within developmental context (Sroufe, 1990; Wakschlag et al., 2010). Our dimensional spectrum approach goes beyond a focus on extreme behaviors to characterization of domains of behavior along a continuum dimensional spectrum that considers context and frequency. The psychometric validation of MAPS‐INT dimensions presented here is a natural outgrowth of our prior work on dimensionalization of early childhood irritability and other externalizing behaviors (Wakschlag et al., 2014; Nili et al., 2023), as well as characterization of the clinical phenomenology and observed features demarcating expression of internalizing problems in young children (Buss, 2011; Luby et al., 2003). Findings hold promise for generating empirically derived parameters for distinctions between what are likely to be normative variations versus extremes of such and identification of pathognomonic “red flags” that are clinical markers. This advances application of the science of “when to worry” framework to young children's anxious and depressive behaviors. As we and others have noted (Bufferd et al., 2023; Wakschlag et al., 2018), thresholds of risk vary based on the nature of the behavior. This is also demonstrated across the multiple cross‐age papers in this special issue validating the MAPSL‐TL Infant‐Toddler and Youth versions (Alam et al., 2023; Hirsch et al., 2023; Kirk et al., 2023; Wiggins, Ureña Rosario, MacNeill et al., 2023). This suggests that, unlike the uniform subjective threshold approach for DSM symptoms, different cut‐points could be useful when demarcating developmental thresholds of severity for internalizing behaviors in this age period, depending on the behavior in question. Most crucial in this regard is the distinction between higher frequency behaviors that most children do which become atypical when done very frequently (e.g., daily or more) versus pathognomonic behaviors that do not reflect normative misbehaviors and are abnormal at lower frequencies (Bufferd et al., 2023; Wakschlag et al., 2018). These patterns were broadly similar across anxious and depressive behaviors, and consistent with prior developmental dimensional work in this domain (Bufferd et al., 2023). They are also consistent with patterns we have previously shown for a broad range of disruptive behavior dimensions (including irritability and low concern for others, although specifics vary by behavior of interest (Wakschlag et al., 2018; see also Alam et al., 2023; Hirsch et al., 2023; Kirk et al., 2023; Wiggins, Ureña Rosario, MacNeill et al., 2023).

The Anxious Behaviors item pool yielded a two‐factor pattern of Fearful‐Worried and Separation Distress Behaviors. In general, Separation Distress behaviors were more normatively occurring than Fearful‐Worried behaviors. For example, more than half of the separation distress behaviors occurred regularly in most children, whereas that was true for only 2 out of 25 fearful‐worried behaviors. Consistent with the developmental specification framework, those anxious behaviors that occurred in developmentally expectable contexts (e.g., *clinginess when separating in unfamiliar context*) were reported in nearly 2/3 of children, whereas those that occurred in developmentally unexpectable contexts (e.g., *fearful‐worried during fun, routine activities or with children*) were reported in less than a quarter of children. Dysregulated, anxious behaviors were also more severe as theorized (e.g., *inconsolable, persists with reassurance*). Of note was the distribution of freezing behavior, due to its centrality in temperament literature on behavioral inhibition. Freezing exhibited a pattern consistent with the pathognomonic expression described above (i.e., ∼87% of children were described as never freezing over the past month, and the frequency thresholds was several times per week) versus many other behaviors whose frequency threshold was more days than not). Even though freezing is a core element of behaviorally inhibited temperament, in research on temperament, it is typically elicited under more extreme laboratory conditions in these studies. This is in contrast to parent report of more naturalistic occurrence here. Contrasting experimental derived behavioral inhibition ratings of observed behavior with parent reports on this dimensional survey, and their differential clinical and predictive utility, will be important in future investigations.

Depressive Behaviors followed a similar pattern. For example, *expressing sad emotions* and *tearfulness during daily routines* was common, whereas *sadness out of the blue* or *sad during fun activities*, *lacking energy*, *being too sad to eat*, and *saying negative things about oneself* occurred less commonly. Importantly, frequency of occurrence that marked abnormality (occurs rarely, i.e., in ≤5% of children) varied by behavior, emphasizing limitations of traditional approaches that apply the same threshold regardless of the nature of the behavior. These varied frequency thresholds based on the nature and context of behavior are especially salient during early childhood, which is a developmental period marked by high emotional intensity and dysregulation in response to challenges. This will be important to account for in future work designed to create clinical cut‐points. Conversely, this is consistent with our prior and current work in irritability with the MAPS‐TL (Wakschlag et al., 2012; Alam et al., 2023; Hirsch et al., 2023; Kirk et al., 2023; Wiggins, Ureña Rosario, MacNeill et al., 2023). That is, *daily* occurrence of depressive and anxious behaviors is rare regardless of behavior type and may serve as a clinical marker for brief screening.

The MAPS‐INT dimensional approach holds promise for examination of normal:abnormal processes in a more fine‐grained manner than symptom count indices or categorical classifications allow. While frameworks like the NIMH Research Domain Criteria (RDoC) advance dimensional approaches conceptually (Cuthbert, 2014), clinical translation has been impeded by the absence of tools that operationalize it. The central contribution of the MAPS‐INT is dimensional characterization of the normal:abnormal spectrum of anxious and depressive behaviors in young children. It translated nuanced features previously requiring intensive assessments into a pragmatic survey tool (Morris et al., 2020). The MAPS toolkit, encompassing the MAPS‐INT for Anxious and Depressive behaviors, the MAPS‐TL for irritability and additional disruptive behavior and attention dysregulation dimensions, now provides full dimensional coverage of the common and modifiable internalizing/externalizing syndromes of early childhood. Such a developmentally‐based, dimensional toolkit has potential value not only in individual assessment, but in large‐scale epidemiological studies such as the NIH‐sponsored Environmental Child Health Outcomes (ECHO) consortium, which strive to capture unfolding patterns in close proximity to exposure (Blackwell et al., 2018).

Indeed, the advent of such dimensional scales provides an opportunity to place internalizing risk along a continuum and to track intervention progress. The full dimensional MAPS‐INT can be deployed as a comprehensive assessment tool in efforts to advance earlier identification and prevention, particularly by identifying young children in “gray areas” of risk. An important next step would be to generate a pragmatic screener from the MAPS‐INT dimensions as has been done in other papers in this special issue (Alam et al., 2023; Hirsch et al., 2023; Kirk et al., 2023; Wiggins, Ureña Rosario, Zhang et al., 2023). Elevations on the scale would point to the need for a more in‐depth clinical evaluation assessing DSM‐5 syndromes and impairment, including gold standard clinical interview measures such as the PAPA (Egger & Angold, 2006). In turn, earlier identified children can receive preventive intervention and/or receive diagnoses and treatment as indicated, as DSM‐based internalizing syndromes and their treatments are well validated in early childhood (Egger & Angold, 2006; Luby, 2013).

### 4.1 Limitations

We leveraged an existing diverse but non‐representative sample to initially establish the dimensional spectra of these internalizing domains. *The trade‐offs inherent in relying on extant data for a validation study introduce several limitations*: (1) Sample size: While the sample was SES‐ and racially/ethnically diverse, we lacked the power to test for method invariance across these sub‐groups as well as by child gender. (2) Coverage across the broad span of early childhood (young toddlers‐preschool age). We originally considered whether subsets of items would need to vary for sub‐periods of early childhood (e.g., at the transition to toddlerhood in second year of life vs. older preschoolers). However, we ultimately decided to deploy a single version that spanned the full early childhood period. In balance, we felt this was developmentally sound and well aligned with pragmatic considerations that have guided the well‐validated PROMIS scales and their developmental applications (Blackwell et al., 2020; Cella et al., 2022). We did this toward our broader goal of real‐world application (i.e., that a single version across early childhood would have greater feasibility). During the item generation period, we also did not identify many items that would necessitate different age‐based versioning, based on clinical and conceptual considerations. While the excellent fit across this broad span of early childhood is encouraging, examination of fit and severity spectrum across these distinct developmental periods within early childhood as well as testing for benefits of age‐graded cut‐points in future large‐scale validation in studies designed for this purpose a priori will be informative. (3) Reliance on a single parent informant is also a limitation. Clinical and predictive validation with multi‐method approaches including direct observation and clinical interviews is an important next step.

*The second set of limitations derives from the structure of the measure itself.* (1) Characterization of non‐irritable depression. The Depressive Behaviors Scale included only non‐irritable indicators, because irritability is conceptualized transdiagnostically within the MAPS framework and is validated as a distinct Temper Loss dimension (Wakschlag et al., 2012; also Alam et al., 2023; Hirsch et al., 2023; Kirk et al., 2023; Wiggins, Ureña Rosario, MacNeill et al., 2023). While irritability is a common symptom of depression, it is also highly non‐specific and therefore cannot be used as a reliable marker of depression in early childhood as distinct from other psychiatric disorders. This has been empirically explored and documented in a large‐scale study of preschool depression (Luby et al., 2003) Further, it has been suggested that the inclusion of irritability in mood and behavioral syndromes blurs their distinctions (Sterba et al., 2007). Follow‐on studies may deploy cluster‐based and/or person‐centered approaches that independently and jointly consider MAPS‐INT anxious and depressive behaviors and MAPS‐TL irritable behaviors for specifying developmentally informed clinical phenomenology in a cross‐cutting manner. (2) Bias introduced by retrospective recall. The MAPS scales use of objective, rather than subjective, recall was designed to reduce subjectivity and improve accuracy of reporting. However, the use of retrospective recall inherently introduces bias. Fortunately, the pioneering work of Buffered, Olino and Dougherty, which uses real time diary methods to assess daily frequency, provides a complementary methodology to examine this question (Bufferd et al., 2017, 2019, 2023). Ideally, a future study could deploy both the MAPS‐INT survey and daily diary methods for the same period to rigorously examine precision trade‐offs. This would enable determination of whether the real time assessment significantly increases precision. This information can then be weighted as part of the selection process for deploying developmental internalizing measures that are dimensional, account for context, and are objective frequency‐based in early childhood populations. The optimal method may vary based, in part, on the nature of study design and outcomes (e.g., in‐depth mechanistic study, epidemiologic study, clinical use). For example, if the daily diary method adds statistically significant precision of estimation due to reduced recall bias, this must be balanced against the more pragmatic survey method for feasibility for routine clinical use.

### 4.2 Conclusion

Negative affectivity has long played a central role in developmental studies of early temperament, and as a substrate of both internalizing and externalizing psychopathology, in largely disparate lines of inquiry (Bates et al., 1998; Buss & Goldsmith, 1998; Cole et al., 2008; Dougherty et al., 2010; Rapee & Coplan, 2010). Temperament measures have strength in characterizing a broad range of normative variation but have typically had less coverage of extremes and were not designed to demarcate atypicalities. In contrast, psychopathology‐oriented scales have abnormal expression as their focus (including several that are developmentally derived, e.g., Carter et al., 2003), but provide less systematic coverage of the range of normative variation. The MAPS‐INT dimensional scales are intended to bridge development and clinical domains to provide a developmentally‐informed contextualized characterization of “when to worry” about young children's anxious and depressive behaviors in a pragmatic fashion. Key advances are nuanced characterization of developmental expression and accounting for context.

The MAPS‐INT internalizing dimensions add to the burgeoning toolkit that operationalizes neurodevelopmental conceptualizations of the early phase of the clinical sequence. The expansion of the developmental specification approach, previously validated for externalizing behaviors, to internalizing behaviors further grounds the translation of emergent clinical risk into developmentally specified terms beginning in the first years of life. This is an important contribution paving the way for earlier identification of clinical risk prior to onset of clinical symptoms with the promise for scaling up to broad application.

## AUTHOR CONTRIBUTIONS

**Lauren S. Wakschlag:** Conceptualization; funding acquisition; investigation; methodology; writing – original draft; writing – review and editing. **Phillip Sherlock:** Conceptualization; formal analysis; investigation; methodology; writing – original draft; writing – review and editing. **Courtney K. Blackwell:** Conceptualization; investigation; methodology; writing – original draft; writing – review and editing. **James L. Burns:** Data curation; formal analysis. **Sheila Krogh‐Jespersen:** Conceptualization; writing – review and editing. **Richard C. Gershon:** Funding acquisition; writing – review and editing. **David Cella:** Conceptualization; funding acquisition; investigation; methodology; writing – review and editing. **Kristin A. Buss:** Conceptualization; investigation; methodology; writing – original draft; writing – review and editing. **Joan L. Luby:** Conceptualization; investigation; methodology; writing – original draft; writing – review and editing.

## CONFLICT OF INTEREST STATEMENT

The authors have no known conflicts of interest to disclose.

## Supporting information

Supplementary Information S1

## Data Availability

The data that support the findings of this study are available from the corresponding author upon reasonable request.
