# Supplementary material for: Modeling the normal:abnormal spectrum of early childhood internalizing behaviors: A clinical‐developmental approach for the Multidimensional Assessment Profiles Internalizing Dimensions
Source: Int J Methods Psychiatr Res. 2023 Oct 10;32(Suppl 1):e1987. doi: 10.1002/mpr.1987 (PMC10654833; doi:10.1002/mpr.1987)
Supplement: Supplementary file 1 — Supplementary Information S1 [file MPR-32-e1987-s001.docx]

**Supplementary Tables**

**Table S1:**

*Comparison of W2W Survey Sub-Study Participant and Non-Participant Demographics*

|  | W2W Survey sub- sample (n=183) | Remaining W2W sample (n=173) | T | df | p |
| --- | --- | --- | --- | --- | --- |
| Child age in months, mean (std. dev.) | 14.0 (1.6) | 14.2 (1.7) | .79 | 354 | .431 |
|  |  |  |  |  |  |
|  | Freq. (%) | Freq. (%) | χ^2^ | df | p |
| Child gender: females | 88 (48.1) | 76 (43.9) | .62 | 1 | .432 |
| Child race/ethnicity |  |  |  |  |  |
| Hispanic | 36 (19.8) | 56 (32.4) | 14.07 | 3 | .003 |
| Non-Hispanic Black | 35 (19.2) | 45 (26.0) |  |  |  |
| Non-Hispanic White | 92 (50.5) | 57 (32.9) |  |  |  |
| Non-Hispanic Other* | 19 (10.4) | 15 (8.7) |  |  |  |
|  |  |  |  |  |  |
| Poverty status**: % Poor | 24 (13.3) | 46 (27.1) | 10.29 | 1 | .001 |
|  |  |  |  |  |  |
| Mother’s education level |  |  |  |  |  |
| High school, GED, or less | 5 (2.7) | 22 (13.9) | 25.97 | 4 | <.001 |
| Associate's degree/Trade School | 12 (6.6) | 20 (12.7) |  |  |  |
| Some college (no degree) | 30 (16.4) | 34 (21.5) |  |  |  |
| Bachelor's degree | 60 (32.8) | 43 (27.2) |  |  |  |
| Graduate degree | 76 (41.5) | 39 (24.7) |  |  |  |

***** Other category includes native American/Alaskan native, Asian, and multiracial.

******Poverty determined by calculating family income-to-needs ratio, which defines poverty status via federal income thresholds by household size (Barajas, Philipsen, & Brooks-Gunn, 2008).

**Table S2:**

*Distribution of MAPS-INT Anxious Behaviors in W2W Preschool Age Children*

|  | **Frequency of Occurrence Over Past Month (%)** | | | | | | Occurs Normatively in Most (>50%) Children | Abnormal Frequency Threshold  ( <5% endorse) |
| --- | --- | --- | --- | --- | --- | --- | --- | --- |
| Item | Never | < weekly | 1-3 days/week | 4-6 days/week | Daily | Many times each day |  |  |
| **Separation Distress** |  |  |  |  |  |  |  |  |
| Cling when separating from you or other parent in an unfamiliar setting | 35.0 | 35.5 | 15.3 | 8.7 | 3.3 | 2.2 | Yes | Daily |
| Cling even after receiving reassurance when separating from you or other parent | 35.0 | 31.7 | 21.3 | 7.7 | 2.7 | 1.6 | Yes | Daily |
| Cling when separating from you or other parent in a familiar setting | 37.7 | 38.8 | 14.8 | 6.6 | 1.1 | 1.1 | Yes | Daily |
| Cling when separating from you or other parent at home | 40.4 | 31.7 | 19.7 | 4.4 | 2.2 | 1.6 | Yes | 4-6 days/week |
| Act worried when separating from you or other parent | 43.2 | 32.2 | 14.2 | 7.7 | 2.2 | 0.5 | Yes | Daily |
| Become inconsolable when separating from you or other parent in a familiar setting | 55.7 | 26.8 | 9.8 | 4.4 | 3.3 | 0.0 | No | 4-6 days/week |
| Become inconsolable when separating from you or other parent in an unfamiliar setting | 58.5 | 19.7 | 13.7 | 4.9 | 2.2 | 1.1 | No | 4-6 days/week |
| Become inconsolable when separating from you or other parent at home | 59.0 | 24.6 | 11.5 | 2.2 | 1.6 | 1.1 | No | 4-6 days/week |
| Act afraid to stay at birthday party or playdate by themselves  **Fearful-Worried** | 69.9 | 16.9 | 8.7 | 2.7 | 1.6 | 0.0 | No | 4-6 days/week |
| Seem  scared or fearful | 37.2 | 40.4 | 18.6 | 2.7 | 0.5 | 0.5 | Yes | 4-6 days/week |
| Act worried when meeting new people/trying new things | 45.4 | 32.8 | 15.8 | 4.9 | 1.1 | 0.0 | Yes | 4-6 days/week |
| Seem worried | 58.5 | 26.8 | 13.1 | 1.1 | 0.5 | 0.0 | No | 4-6 days/week |
| Wake up at night scared | 60.7 | 25.1 | 11.5 | 2.7 | 0.0 | 0.0 | No | 4-6 days/week |
| Seem fearful or worried when out in public | 61.7 | 28.4 | 8.2 | 1.1 | 0.5 | 0.0 | No | 4-6 days/week |
| Seem nervous | 61.7 | 26.8 | 10.4 | 0.5 | 0.5 | 0.0 | No | 4-6 days/week |
| Seem fearful or worried at daycare, school, or other familiar settings away from home | 63.9 | 26.2 | 8.7 | 0.5 | 0.5 | 0.0 | No | 4-6 days/week |
| Get startled easily | 63.9 | 24.0 | 8.7 | 2.7 | 0.0 | 0.5 | No | 4-6 days/week |
| Remain inconsolable even after receiving reassurance | 65.0 | 27.3 | 6.6 | 0.0 | 0.5 | 0.5 | No | 4-6 days/week |
| Keep worrying even after receiving reassurance | 66.7 | 23.5 | 9.3 | 0.5 | 0.0 | 0.0 | No | 4-6 days/week |
| Get scared really easily | 67.8 | 20.8 | 9.8 | 1.1 | 0.0 | 0.5 | No | 4-6 days/week |
| Seem afraid to engage in things that are fun | 68.3 | 24.0 | 6.6 | 1.1 | 0.0 | 0.0 | No | 4-6 days/week |
| Seem anxious | 69.9 | 20.8 | 7.7 | 1.1 | 0.5 | 0.0 | No | 4-6 days/week |
| Seem fearful or worried at home | 72.7 | 20.8 | 6.0 | 0.5 | 0.0 | 0.0 | No | 4-6 days/week |
| Act worried when out with you or other parent in public | 74.3 | 20.2 | 4.4 | 1.1 | 0.0 | 0.0 | No | 1-3 days/week |
| Seem fearful or worried at bedtime | 73.8 | 16.4 | 7.1 | 2.2 | 0.5 | 0.0 | No | 4-6 days/week |
| Seem fearful or worried during fun activities | 74.9 | 21.3 | 3.8 | 0.0 | 0.0 | 0.0 | No | 1-3 days/week |
| Seem tense | 76.0 | 19.1 | 4.9 | 0.0 | 0.0 | 0.0 | No | 1-3 days/week |
| Act worried when in a group of children | 78.1 | 13.1 | 7.1 | 1.6 | 0.0 | 0.0 | No | 4-6 days/week |
| Worry about what could happen to him/her | 78.1 | 17.5 | 3.8 | 0.5 | 0.0 | 0.0 | No | 1-3 days/week |
| Act scared before daycare/preschool | 79.2 | 13.1 | 5.5 | 0.0 | 1.1 | 1.1 | No | 4-6 days/week |
| Act worried during daily routines, such as bedtime, mealtime, or getting dressed | 80.9 | 13.7 | 5.5 | 0.0 | 0.0 | 0.0 | No | 4-6 days/week |
| Suddenly become anxious "out of nowhere" or for no reason | 86.3 | 9.3 | 3.3 | 1.1 | 0.0 | 0.0 | No | 1-3 days/week |
| Freeze because he or she was so scared | 86.9 | 10.9 | 1.1 | 1.1 | 0.0 | 0.0 | No | 1-3 days/week |
| Become uncontrollably distressed when anxious | 90.2 | 8.2 | 1.6 | 0.0 | 0.0 | 0.0 | No | 1-3 days/week |

**Table S3:**

*Distribution of MAPS-INT Depressive Behaviors in W2W Preschool Age Children*

|  | **Frequency of Occurrence Over Past Month (%)** | | | | | | Occurs Normatively in Most (>50%) Children | Abnormal Frequency Threshold  ( < 5% endorse) |
| --- | --- | --- | --- | --- | --- | --- | --- | --- |
|  | Never | < weekly | 1-3 days/week | 4-6 days/week | Daily | Many times each day |  |  |
| **Depressive Behaviors** |  |  |  |  |  |  |  |  |
| Seem sad | 29.5 | 45.9 | 20.2 | 2.7 | 1.1 | 0.5 | Yes | 4-6 days/week |
| Keep crying even when you or other parent tried to comfort him/her | 30.6 | 51.9 | 14.8 | 1.6 | 1.1 | 0.0 | Yes | 4-6 days/week |
| Lack enjoyment when interacting with unfamiliar adults | 39.3 | 37.7 | 12.6 | 9.8 | 0.5 | 0.0 | Yes | Daily |
| Get tearful or weepy during daily routines, such as bedtime, mealtime, or getting dressed | 39.3 | 31.7 | 23.0 | 4.4 | 1.6 | 0.0 | Yes | 4-6 days/week |
| Act withdrawn when interacting with unfamiliar adults | 41.0 | 31.7 | 19.7 | 3.3 | 1.6 | 2.7 | Yes | 4-6 days/week |
| Not seem to enjoy activities and play | 49.2 | 42.6 | 6.0 | 0.5 | 0.0 | 1.6 | Yes | 4-6 days/week |
| Seem uninterested in eating food he/she usually likes | 53.0 | 30.1 | 15.3 | 1.1 | 0.0 | 0.5 | No | 4-6 days/week |
| Not seem to enjoy interacting with other children | 55.7 | 36.1 | 7.7 | 0.5 | 0.0 | 0.0 | No | 4-6 days/week |
| Have a hard time having fun | 56.8 | 39.9 | 3.3 | 0.0 | 0.0 | 0.0 | No | 1-3 days/week |
| Not seem interested in doing things he/she usually likes | 59.0 | 35.0 | 6.0 | 0.0 | 0.0 | 0.0 | No | 4-6 days/week |
| Lack enthusiasm | 59.6 | 35.5 | 4.9 | 0.0 | 0.0 | 0.0 | No | 1-3 days/week |
| Not get as excited as you or other parent would expect | 61.2 | 31.7 | 7.1 | 0.0 | 0.0 | 0.0 | No | 4-6 days/week |
| Act sad or gloomy | 62.3 | 28.4 | 8.2 | 1.1 | 0.0 | 0.0 | No | 4-6 days/week |
| Act withdrawn when in a group of children | 62.3 | 26.8 | 9.3 | 1.6 | 0.0 | 0.0 | No | 4-6 days/week |
| Have a hard time enjoying him/herself | 63.4 | 32.8 | 3.8 | 0.0 | 0.0 | 0.0 | No | 1-3 days/week |
| Not seem to enjoy interacting with other familiar adults | 63.9 | 31.1 | 4.4 | 0.5 | 0.0 | 0.0 | No | 1-3 days/week |
| Act withdrawn when out with you or other parent in public | 64.5 | 29.0 | 4.9 | 1.1 | 0.5 | 0.0 | No | 1-3 days/week |
| Not seem to enjoy interacting with you or other parent at home | 67.8 | 30.6 | 1.6 | 0.0 | 0.0 | 0.0 | No | 1-3 days/week |
| Seem withdrawn | 68.3 | 25.7 | 6.0 | 0.0 | 0.0 | 0.0 | No | 4-6 days/week |
| Act mopey throughout the day | 67.8 | 26.2 | 5.5 | 0.5 | 0.0 | 0.0 | No | 4-6 days/week |
| Act withdrawn when interacting with other familiar adults | 69.9 | 24.0 | 4.4 | 1.6 | 0.0 | 0.0 | No | 1-3 days/week |
| Act withdrawn during daily routines, such as bedtime, mealtime, or getting dressed | 74.9 | 20.2 | 4.9 | 0.0 | 0.0 | 0.0 | No | 1-3 days/week |
| Suddenly act sad or gloomy "out of the blue" or for no reason | 75.4 | 16.9 | 6.6 | 0.5 | 0.5 | 0.0 | No | 4-6 days/week |
| Act withdrawn when interacting with you or other parent at home | 77.0 | 20.8 | 2.2 | 0.0 | 0.0 | 0.0 | No | 1-3 days/week |
| Lack energy and seem not to care | 79.8 | 16.4 | 3.3 | 0.5 | 0.0 | 0.0 | No | 1-3 days/week |
| Seem sad when playing with other kids | 82.5 | 15.8 | 1.6 | 0.0 | 0.0 | 0.0 | No | 1-3 days/week |
| Seem too sad to eat | 91.8 | 7.7 | 0.0 | 0.5 | 0.0 | 0.0 | No | 1-3 days/week |
| Say negative things about him/herself | 92.3 | 7.1 | 0.5 | 0.0 | 0.0 | 0.0 | No | 1-3 days/week |

**Supplementary References:**

Barajas, R. G., Philipsen, N., & Brooks-Gunn, J. (2008). Cognitive and emotional outcomes for children in poverty. In D. Crane & T. Heaton (Eds.), *Handbook of Families and Poverty* (pp. 311-333). Sage.
